# Body Composition Changes After Bariatric Surgery: Magnitude, Timing, and Determinants of Excessive Fat-Free Mass Loss

**DOI:** 10.3390/jcm15020630

**Published:** 2026-01-13

**Authors:** Noelia Perez-Romero, Montse Adell Trapé, Assumpta Caixàs, Ariadna Cidoncha Secilla, Christian Jose Herrero Vicente, Marina Luengo Moral, Alba Hernandez-Lazaro, Alexis Luna Aufroy

**Affiliations:** 1 Esophagogastric and Bariatric Unit, General Surgery Department, Parc Taulí Hospital Universitari, 08208 Sabadell, Spain; madell@tauli.cat (M.A.T.); acidoncha@tauli.cat (A.C.S.); cjherrero@tauli.cat (C.J.H.V.); aluna@tauli.cat (A.L.A.); 2 Teaching Unit, Parc Taulí Corporation, Medicine Department, Universitat Autònoma de Barcelona, 08208 Sabadell, Spain; acaixas@tauli.cat; 3 Endocrinology and Nutrition Department, Parc Taulí Hospital Universitari, 08208 Sabadell, Spain; mluengo@tauli.cat (M.L.M.); ahernandezl@tauli.cat (A.H.-L.); 4 Institut d’Investigació i Innovació Parc Taulí (I3PT-CERCA), 08208 Sabadell, Spain

**Keywords:** bariatric surgery, body composition, fat-free mass loss, sarcopenic obesity, weight loss outcomes, bioelectrical impedance analysis, postoperative monitoring

## Abstract

**Background:** Bariatric surgery effectively treats severe obesity, but postoperative weight loss includes reductions in both fat mass (FM) and fat-free mass (FFM). Excessive FFM loss may increase the risk of sarcopenia, frailty, and long-term weight regain, yet its magnitude and determinants are not fully established. **Methods:** We conducted a retrospective analysis of a prospectively collected cohort of 179 patients who underwent laparoscopic or robotic Roux-en-Y gastric bypass between January 2020 and December 2022. Anthropometric parameters and body composition (bioelectrical impedance analysis) were measured preoperatively and at 6 and 12 months. The proportion of FFM loss relative to total weight loss (%FFML/WL) was calculated, and excessive FFM loss was defined using published cut-offs (≥25%, ≥30%, and ≥35%). Predictors of FFM preservation were assessed through stepwise regression. **Results:** Baseline BMI was 44.1 ± 4.6 kg/m^2^, FM 54.6 ± 10.7 kg, and FFM 61.1 ± 11.9 kg. At 6 and 12 months, BMI decreased to 31.0 ± 4.2 and 28.8 ± 4.4 kg/m^2^, respectively; FM decreased to 35.6 ± 11.0 and 22.0 ± 10.0 kg; and FFM to 54.7 ± 9.5 and 50.1 ± 7.0 kg (all *p* < 0.001). Most FFM loss occurred within the first 6 months (mean − 6.4 kg). Median %FFML/WL was 26.4% at 6 months and 28.7% at 12 months. Excessive FFM loss affected 41–46% of patients (≥25%), 27–31% (≥30%), and 14% (≥35%). In multivariable analysis, FFM at 6 months was the only independent predictor of FFM at 12 months (*p* < 0.001). **Conclusions:** Bariatric surgery leads to substantial FM and FFM reductions, with nearly half of patients exceeding established %FFML/WL alert thresholds. Early postoperative body composition monitoring may help identify individuals at higher risk of FFM depletion and guide preventive strategies such as adequate protein intake and resistance training.

## 1. Introduction

Morbid obesity is a global public health problem, associated with high morbidity and mortality and an increased risk of metabolic and cardiovascular diseases. Bariatric surgery is the most effective treatment for severe and morbid obesity, achieving sustained weight loss and improvement in metabolic and cardiovascular comorbidities [1,2].

However, weight loss after surgery does not occur solely at the expense of fat mass (FM), but also involves a significant reduction in fat-free mass (FFM), which includes muscle mass. Preservation of muscle mass is essential, as it plays a key role in energy metabolism, thermoregulation, bone remodelling, and functional capacity. Furthermore, it acts as a reservoir of glycogen, lipids, and proteins [3,4,5]. An excessive decrease in FFM can promote the development of sarcopenia, which is associated with frailty, functional decline, and increased morbidity and mortality [3]. Sarcopenia is defined as the loss of muscle mass accompanied by a decrease in strength or physical performance. When it coexists with obesity, it is called sarcopenic obesity, a condition associated with frailty, disability, falls, fractures, metabolic disturbances, and increased mortality [6]. After bariatric surgery, the risk of developing sarcopenia is increased, with reported prevalence rates rising from 8% preoperatively to 32% at one year [7,8].

Several studies have documented the magnitude of fat-free mass loss after bariatric surgery. A recent meta-analysis showed that, on average, more than 8 kg of fat-free body mass are lost in the first year, with more than 50% of this loss occurring in the first 3–6 months postoperatively [9]. These data reinforce the need to closely monitor changes in body composition during early follow-up.

Beyond its functional impact, fat-free mass (FFM) loss can influence the regulation of energy balance. Recent evidence suggests that a greater proportion of FFM loss during weight reduction is associated with increased appetite and a higher risk of weight regain, especially in men [10]. This finding underscores that preserving muscle tissue is not only relevant for physical capacity but also for the long-term success of bariatric surgery. These phenomena highlight the importance of assessing not only the amount of weight lost but also the quality of that loss.

Several authors have therefore proposed expressing fat-free mass loss as the proportion of total weight loss (%FFML/WL), as an indicator of the “quality” of weight loss. Using this metric, cut-offs in the range of 25–35% of total weight loss derived from FFM have been suggested as alert thresholds for excessive loss of metabolically active tissue [8,10,11].

The aim of this study was to analyse the evolution of body composition after bariatric surgery and to determine the prevalence of excessive FFM loss, as well as the factors associated with its development.

## 2. Materials and Methods

A retrospective study was conducted using a prospective database of patients who underwent laparoscopic or robotic gastric bypass between January 2020 and December 2022 at a tertiary hospital. Pure restrictive techniques, hypoabsorptive techniques, and revision surgeries were excluded. Indications for bariatric surgery followed current international and national guidelines and included severe obesity (BMI ≥ 40 kg/m^2^) or BMI ≥ 35 kg/m^2^ with obesity-related comorbidities, after failure of structured conservative management.

All patients were managed according to the standardized multidisciplinary perioperative care protocol of our center (CSPT), which includes structured preoperative assessment, intraoperative management, and postoperative follow-up. Patients underwent a comprehensive evaluation including nutritional, endocrinological, psychological, pneumological (sleep apnea screening), and surgical assessment. Preoperative laboratory testing, ECG, chest X-ray, pulmonary function tests, polysomnography when indicated, and abdominal ultrasound were routinely performed. Comorbidities were optimized prior to surgery, including hypertension, type 2 diabetes, dyslipidemia, and obstructive sleep apnea, with mandatory CPAP use for at least six weeks in patients with SAHS. Two dedicated nutritional consultations were performed to assess dietary adherence and provide postoperative dietary instructions. Patients also participated in structured educational sessions addressing postoperative diet progression, hydration, supplementation, and behavioural changes. After surgery, patients were followed according to a structured schedule by Nutrition, Endocrinology, Surgery, and Psychology. Follow-up visits included dietary progression, evaluation of tolerance to protein intake, monitoring for nutritional deficiencies, weight and body composition tracking, and reinforcement of physical activity recommendations. As part of standard postoperative care, patients were encouraged to progressively increase physical activity. However, no structured or supervised resistance training program was systematically implemented, and physical activity was not objectively assessed. Supplementation with vitamins, minerals, calcium–vitamin D, and iron was prescribed according to standardized protocols.

Demographic, anthropometric, and body composition data were collected at three time points: preoperatively, at 6 months, and at 12 months. Body composition was assessed using a multifrequency bioelectrical impedance analyzer (Tanita Corporation, Tokyo, Japan, Tanita^®^), model used according to institutional protocol). Quantitative variables were expressed as mean ± standard deviation (SD) or median and interquartile range (IQR), depending on their distribution. Changes in body weight, body mass index (BMI), fat mass (FM), and fat-free mass (FFM) were calculated between preoperative, 6 months, and 12 months postoperatively. Fat mass (FM) and fat-free mass (FFM) were obtained from multifrequency bioelectrical impedance analysis, following standard definitions used in bariatric and body composition research. The proportion of fat-free mass loss relative to total weight loss (%FFML/WL) was calculated as: %FFML/WL = (FFM loss ÷ total weight loss) × 100 for each interval. In line with previously published work, this index was used to characterize the “quality” of weight loss, with higher values indicating poorer FFM preservation [8,10]. Excessive FFM loss was defined using established cut-off points of ≥25%, ≥30%, and ≥35% %FFML/WL, to allow comparability with prior studies [10]. The prevalence of each of these thresholds was calculated at 6 and 12 months postoperatively. Although total body water and phase angle were available from bioelectrical impedance analysis, these parameters were not included in the present analysis, as this study focused on longitudinal changes in fat mass and fat-free mass, in accordance with the predefined objectives and previous bariatric literature. 

Artificial intelligence tools were used solely for language editing and/or grammar checking. The authors take full responsibility for the content of the manuscript.

### Statistical Analysis

The comparison of means between time points was performed using the paired Student’s *t*-test, or equivalent non-parametric tests in the case of non-normal distributions. Associations between fat-free mass (FFM) loss and demographic factors (age, sex, baseline BMI) were also explored using bivariate analysis. Associations between %FFML/WL and key baseline variables (including age, sex, and baseline BMI) were explored using bivariate analyses. Statistical analysis was performed using IBM SPSS Statistics for Windows, version 20.0 (IBM Corp., Armonk, NY, USA). A *p*-value < 0.05 was considered statistically significant.

## 3. Results

A total of 179 patients were included, of whom 139 were women (77.7%), with a mean age of 48.7 ± 8.8 years and a baseline BMI of 44.1 ± 4.6 kg/m^2^. Baseline body composition showed a mean fat mass (FM) of 54.6 ± 10.7 kg and a fat-free mass (FFM) of 61.1 ± 11.9 kg (Table 1).

**Figure 1:**
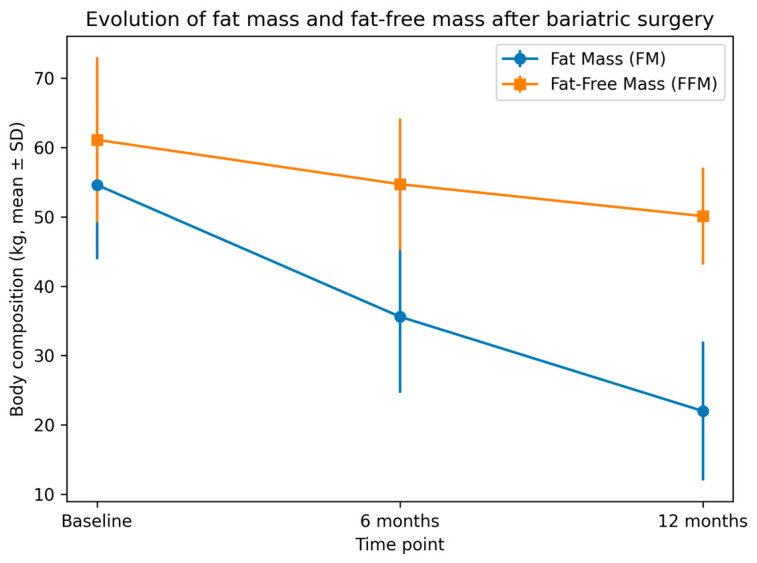
Evolution of fat mass (FM) and fat-free mass (FFM) at baseline, 6 months, and 12 months after bariatric surgery. Values are expressed as mean ± standard deviation (SD). Fat mass decreased markedly and continuously during the first postoperative year, whereas fat-free mass showed an early decline followed by relative stabilization between 6 and 12 months.

1. Weight loss trajectory. Body weight decreased significantly and progressively throughout follow-up (Table 2). At 6 months, patients had lost an average of 30.0 kg (SD 8.5), corresponding to a reduction in BMI to 31.0 ± 4.2 kg/m^2^. At 12 months, mean total weight loss reached 42.0 kg (SD 10.2), with BMI decreasing to 28.8 ± 4.4 kg/m^2^ (all *p* < 0.001 vs. baseline). When expressed as total weight loss percentage (%TWL), patients lost on average: 29.3% TWL at 6 months, and 35.9% TWL at 12 months. These values are consistent with expected weight trajectories after Roux-en-Y gastric bypass.

2. Body composition evolution. Body composition changes mirrored the weight-loss pattern, with substantial reductions in FM and more modest but clinically relevant declines in FFM (Table 2 and Figure 1). FM decreased steadily throughout follow-up, falling from 54.6 ± 10.7 kg at baseline to 35.6 ± 11.0 kg at 6 months and 22.0 ± 10.0 kg at 12 months. FFM loss occurred predominantly during the first 6 months, when FFM dropped by 6.4 kg on average, reaching 54.7 ± 9.5 kg. Between 6 and 12 months, the additional decline was smaller (mean FFM at 12 months: 50.1 ± 7.0 kg), consistent with the early and preferential mobilization of fat-free mass observed after major weight reduction procedures. When expressed relative to total weight loss, FFM accounted for a median of 26.4% (IQR 21.0–32.8) of overall weight loss at 6 months and 28.7% (IQR 23.1–35.2) at one year (Table 2). Thus, although most patients preserved the majority of their fat-free mass, FFM contributed to approximately one quarter of total weight lost during the first postoperative year.

3. Prevalence and severity of excessive FFM loss. To contextualize the clinical relevance of FFM depletion, the proportion of weight loss attributable to FFM (%FFML/WL) was stratified according to cut-offs proposed by Nuijten et al. Higher proportions reflect poorer fat-free mass preservation.

- ≥25% FFML/WL → moderate alert;
- ≥30% FFML/WL → high fat-free mass loss;
- ≥35% FFML/WL → excessive fat-free mass loss, clinically concerning.

Applying these thresholds, the prevalence of excessive FFM loss (%FFML/WL) was relevant (Table 3): at 6 months, 41% of patients exceeded the 25% threshold, 27% surpassed 30%, and 13.4% exhibited severe FFM loss (≥35%). Prevalence increased slightly at 12 months, with 46%, 31%, and 14.5%, respectively, meeting these cut-off criteria (Table 3). Notably, around one in seven patients experienced excessive fat-free mass loss at one year, despite overall successful weight outcomes.

4. Subgroup analysis. A stepwise linear regression analysis was performed to identify predictors of fat-free mass (FFM) at 12 months. FFM at 6 months was the only variable retained in the model. No other demographic or anthropometric variables—including sex, age, baseline BMI, baseline FFM, or total weight loss at 6 or 12 months—met inclusion criteria for the model. In exploratory bivariate analyses, sex was not significantly associated with %FFML/WL or with FFM preservation. The regression model showed an excellent fit, explaining 79.2% of the variance in FFM at 12 months (R = 0.890; R^2^ = 0.792; adjusted R^2^ = 0.777), with a standard error of the estimate of 2.72 kg. The ANOVA confirmed the statistical significance of the model (F = 53.18, *p* < 0.001). FFM at 6 months was strongly associated with postoperative FFM (B = 1.193, SE = 0.164, *p* < 0.001), indicating that patients with higher fat-free mass at 6 months tended to preserve more fat-free mass at one year. No other demographic or anthropometric variables—including age, sex, baseline BMI, baseline FFM, or total weight loss at 6 or 12 months—met inclusion criteria for the model.

## 4. Discussion

In our cohort of 179 patients undergoing gastric bypass, we observed significant weight loss at one year of follow-up, with a mean BMI of 28.3 kg/m^2^ and a total weight loss of 35.9%. Weight loss occurred at the expense of both fat mass (FM) and fat-free mass (FFM), with a mean reduction of 9.7 kg of FFM in one year. The loss of FFM was more pronounced during the first six months, consistent with what has been described in the literature [9,11,12,13]. In our study, the median %FFML/WL was 26.4% at 6 months and 28.7% at one year, figures comparable to those previously reported (17–31% of weight loss at the expense of FFM).

Beyond mean values, the distribution of patients according to clinically relevant cut-off points provides additional insight into the magnitude of and variability in postoperative FFM loss. In our cohort, up to 46% of patients exceeded the 25% FFML/WL threshold at one year, 31% surpassed 30%, and 14.6% reached ≥35%, a level considered excessive and clinically concerning. These proportions are in line with the findings of Nuijten et al. [10], who reported a prevalence of 14–46% depending on the time point and threshold applied. Importantly, our data confirm that a relevant minority of patients experience disproportionately high fat-free mass loss despite otherwise successful weight outcomes, highlighting the clinical utility of %FFML/WL as a phenotypic marker to identify individuals at greater risk.

As in the cohort described by Nuijten et al., in our analysis age, sex, and baseline BMI did not show a significant association with the magnitude of FFM loss. However, several considerations help contextualise this finding. First, our sample consisted predominantly of women (>75%), reducing sex-related variability. Second, we included exclusively patients undergoing Roux-en-Y gastric bypass, whereas individuals with more extreme BMI values, older age, or higher surgical risk profiles are more frequently selected for sleeve gastrectomy as a first-stage or safer alternative procedure in our centre. This selection pattern likely narrowed the variability in baseline characteristics, thereby limiting the ability to detect associations present in more heterogeneous cohorts. Taken together, these differences underscore the importance of considering procedure type and population characteristics when comparing studies.

The clinical importance of postoperative fat-free mass loss lies in the central physiological role of FFM, particularly skeletal muscle mass, in maintaining basal metabolic rate, functional capacity, bone health, and metabolic control. It has been shown that for every kilogram of FFM lost after bariatric surgery, resting energy expenditure decreases significantly—an effect greater than that observed after calorie-restricted diets alone [4]. Moreover, a greater proportion of FFM loss has been associated with increased appetite and a higher risk of weight regain [10], potentially compromising the long-term success of bariatric surgery. From this perspective, a %FFML/WL above the 30–35% range identifies a subgroup of patients with a potentially adverse metabolic trajectory, who may benefit from closer monitoring and earlier targeted interventions. Our use of the 25–35% range as alert thresholds is consistent with the approach proposed by Nuijten et al., facilitating direct comparison of prevalence estimates across studies.

Our findings should also be interpreted in the context of the growing interest in sarcopenic obesity. After bariatric surgery, the prevalence of sarcopenia has been reported to increase from 8% preoperatively to 32% at one year [7,8], with relevant clinical implications: increased risk of frailty, falls, fractures, functional disability, and metabolic complications. The overlap of obesity and sarcopenia can have negative synergistic effects, favouring the development of cardiovascular and metabolic comorbidities. In our cohort, we did not assess muscle strength or physical performance; so, we were unable to diagnose sarcopenia according to current criteria, which is a significant limitation.

The pathophysiology of postoperative fat-free mass loss is complex. Intense caloric restriction, insufficient early protein intake (around 30 g/day vs. the recommended 60–80 g/day), limited tolerance of 20–40 g of protein per meal, physical inactivity during recovery, and the absence of protein storage lead to skeletal muscle being used as an amino acid source. Hormonal alterations—including reduced leptin, insulin and ghrelin, and increased PYY and adiponectin—also contribute to these changes [14,15,16,17]. In addition, hydration changes and shifts in the metabolically active components of FFM may influence measurements and tissue behaviour during weight loss, as previously described [5], further complicating the assessment of body composition. Bone remodelling alterations may also increase the risk of osteosarcopenia after surgery.

Clinically, these findings support the need for systematic monitoring of body composition from the preoperative period throughout postoperative follow-up, especially during the first six months, which constitute the critical window of greatest risk for FFM loss. Early detection of patients with a high proportion of FFML should guide targeted interventions aimed at preserving muscle mass: optimisation of protein intake (≥60 g/day or 1.1–1.5 g/kg ideal body weight), adequate distribution throughout the day, specific supplementation when required, and early progressive resistance training. Although evidence regarding the efficacy of these interventions remains limited and inconsistent, a combined strategy integrating nutrition and exercise appears reasonable.

Limitations of our study include its retrospective nature, the significant loss to follow-up, and the absence of functional parameters of sarcopenia (strength and physical performance). Additionally, although bioelectrical impedance has been validated against DXA, it may underestimate or overestimate fat mass in individuals with severe obesity or hydration fluctuations. Nonetheless, the use of the same technique for all measurements and the sample size provide robustness to the findings. In addition, TBW and phase angle were not analysed, and future prospective studies incorporating these measures may help refine the interpretation of BIA-derived FFM changes after bariatric surgery. Finally, the lack of objective assessment of physical activity and the absence of a structured resistance training program represent limitations, as these factors may influence postoperative fat-free mass preservation.

Our results reinforce the evidence that bariatric surgery is associated with substantial weight loss, but also highlight that a non-negligible proportion of patients experience excessive fat-free mass loss, a phenomenon with important metabolic and functional implications that warrants proactive identification and management.

## 5. Conclusions

Bariatric surgery induces substantial weight loss, accompanied by a progressive reduction in both fat mass and fat-free mass. Beyond confirming this well-described phenomenon, our study provides clinically relevant information on the magnitude and timing of and variability in fat-free mass loss, using the proportion of fat-free mass loss relative to total weight loss (%FFML/WL) as an indicator of weight-loss quality. In our cohort, nearly half of the patients exceeded the 25–30% FFML/WL alert thresholds, and approximately 15% reached excessive fat-free mass loss (≥35%) within one year, underscoring the high prevalence and clinical relevance of disproportionate fat-free mass loss after bariatric surgery.

The absence of associations with age, sex or baseline BMI in our series—composed predominantly of women and limited to Roux-en-Y gastric bypass—suggests that postoperative fat-free mass loss may be driven by factors beyond baseline demographic characteristics. Moreover, the strong predictive value of FFM at 6 months highlights the early postoperative period as the critical window for intervention.

Routine assessment of body composition, together with strategies to optimize protein intake and promote early progressive resistance training, should be prioritised to mitigate the risk of sarcopenia and sarcopenic obesity after bariatric surgery. Future research should refine clinically meaningful FFML thresholds and develop standardised multimodal interventions aimed at improving long-term metabolic and functional outcomes.

## Figures and Tables

**Table 1:** Baseline characteristics of the study cohort, overall and stratified by sex. Values are expressed as mean ± standard deviation (SD). BMI (body mass index).

**Variable**	**Total (n = 179)** **Mean ± SD**	**Women (n = 139)** **Mean ± SD**	**Men (n = 40)** **Mean ± SD**
Age (years)	48.7 ± 8.8	48.5 ± 9.1	49.5 ± 8.0
Body weight (kg)	118.6 ± 19.8	115.5 ± 14.2	132.6 ± 18
Preoperative BMI (kg/m^2^)	44.1 ± 4.6	44.4 ± 4.6	43.3 ± 4.6
Fat mass (kg)	54.6 ± 10.7	56.0 ± 10.1	50.1 ± 11.7
Fat-free mass (kg)	61.1 ± 11.9	55.8 ± 6.6	78.3 ± 8.8

**Table 2:** Evolution of body weight and BMI after bariatric surgery. Values are expressed as mean ± standard deviation (SD) or median (interquartile range, IQR). Comparisons with baseline performed with paired Student’s *t* test. FFML/WL: percentage of fat-free mass loss relative to total weight loss. “–”: not applicable.

**Variable**	**N**	**Baseline** **(Mean ± SD)**	**6 Months** **(Mean ± SD)**	**12 Months** **(Mean ± SD)**	***p*** **-Value** **(vs. Baseline)**
Body weight (kg)	179	118.6 ± 19.8	83.6 ± 15.7	77.2 ± 14.5	<0.001
BMI (kg/m^2^)	179	44.1 ± 4.6	31.0 ± 4.2	28.8 ± 4.4	<0.001
Total weight loss (%)	179	–	29.3 ± 6.1	35.9 ± 8.5	<0.001
Fat mass (kg)	179	54.6 ± 10.7	35.6 ± 11.0	22.0 ± 10.0	<0.001
Fat-free mass (kg)	179	61.1 ± 11.9	54.7 ± 9.5	50.1 ± 7.0	<0.001
%FFML/WL (fat-free mass loss/weight loss)	–	–	26.4% (IQR 21.0–32.8)	28.7% (IQR 23.1–35.2)	–

**Table 3:** Prevalence of excessive FFM loss depending on cut-off criteria.

**Cut-Off (%FFML/WL)**	**6 Months (%, n/N)**	**12 Months (%, n/N)**
≥25%	41% (73/179)	46% (82/179)
≥30%	27% (48/179)	31% (55/179)
≥35%	13.4% (24/179)	14.5% (26/179)

## Data Availability

The data presented in this study are not publicly available due to ethical and privacy restrictions, as they contain confidential clinical information. De-identified data may be provided by the corresponding author upon reasonable request and with permission from the institutional ethics committee.

## Abbreviations

The following abbreviations are used in this manuscript:

BIA	Bioelectrical Impedance Analysis
BMI	Body Mass Index
CPAP	Continuous Positive Airway Pressure
ECG	Electrocardiogram
FFM	Fat-Free Mass
FM	Fat Mass
%FFML/WL	Percentage of Fat-Free Mass Loss relative to Total Weight Loss
GI	Gastrointestinal
IQR	Interquartile Range
kg	Kilogram
kg/m^2^	Kilograms per Square Meter
RYGB	Roux-en-Y Gastric Bypass
SAHS	Sleep Apnea–Hypopnea Syndrome
SD	Standard Deviation
TWL	Total Weight Loss
ΔWeight	Change in Total Body Weight
ΔFFM	Change in Fat-Free Mass
